# Marked Multiple Tendinitis at the Onset of Rheumatoid Arthritis in a Patient with Heterozygous Familial Hypercholesterolemia: Ultrasonographic Observation

**DOI:** 10.1155/2014/486348

**Published:** 2014-06-09

**Authors:** Takeshi Suzuki, Akiko Okamoto

**Affiliations:** Division of Rheumatology, Mitsui Memorial Hospital, 1 Kandaizumi-cho, Chiyoda-ku, Tokyo 101-8643, Japan

## Abstract

A 59-year-old woman who had been diagnosed with heterozygous familial hypercholesterolemia developed rheumatoid arthritis (RA). She presented with marked tendinitis of the Achilles tendons, patellar tendons, and finger extensor tendons at the onset of RA. Ultrasonographic examination revealed that tendon lesions were predominantly tendinitis rather than paratenonitis, and that the tendinitis was of the noninsertional variety, rather than the insertional variety. Preexisting tendon xanthomas might have contributed to the unusually dominant noninsertional tendinitis of multiple tendons.

## 1. Introduction

The prevalence of rheumatoid arthritis (RA) is relatively constant at 0.5 to 1.0% in many populations [1]. The estimated prevalence of RA is 1.0% of the entire Japanese population aged ≧16 to <75 years [2]. Familial hypercholesterolemia (FH) is a genetic disorder associated with severe hypercholesterolemia, atherosclerosis, and xanthomas at various sites in which the primary defect is a mutation in the low-density lipoprotein receptor [3, 4]. Although homozygous FH is a very rare disease with a poor prognosis, heterozygous FH (HeFH) is a relatively common disorder with a prevalence of approximately 0.2% in most countries, including Japan [5]. The frequency of the incidental association of HeFH and RA is estimated to be around one per 50,000 adults. Despite this, only one case has been reported in the English literature [6]. Herein, we report a case of HeFH presenting with marked multiple tendinitis at the onset of RA.

## 2. Case Presentation

A 59-year-old Japanese woman was referred to our hospital in May 2012 due to posterior ankle pain and anterior knee pain in both legs. She had been diagnosed with hypertension and HeFH at the age of 57. At that time, her total cholesterol was 458 mg/dL (11.8 mmol/L), triglycerides 237 mg/dL (2.67 mmol/L), low-density lipoprotein cholesterol (LDL-C) 359 mg/dL (9.4 mmol/L), and high-density lipoprotein cholesterol 45 mg/dL (1.18 mmol/L). She had a family history of hypercholesterolemia and premature coronary heart disease (CHD) but had no family history of RA. She had no past history of CHD but had a severe stenosis of the left internal carotid artery. She had been treated with 2 mg of pitavastatin for two years and 10 mg of ezetimibe for six months at the time of her initial visit to our hospital. Her serum LDL-C level was reduced to around 260 mg/dL (6.7 mmol/L) by the administration of pitavastatin and was further reduced to around 170 mg/dL (4.4 mmol/L) by adding ezetimibe. Her cholesterol levels had been stable for the last six months.

She had experienced pain in the bilateral Achilles tendons for six weeks and in the bilateral knees and the metacarpophalangeal (MCP) joint of the left third finger for four weeks at the time of her initial visit to our hospital. Physical examination revealed bilateral arcus cornea. Swelling and thickening of the bilateral Achilles tendons were observed along the midportion of the tendon. There was tenderness on palpation along the Achilles tendons from the midportion to the distal insertion into the calcaneal bone. There was also tenderness on palpation of the bilateral tibial tuberosities and the right medial epicondyle of the humerus. As for small joints, there was tenderness on palpation of the first and fifth metatarsophalangeal (MTP) joints of the right foot and the second MTP joint of the left foot. The dorsal aspect of the third MCP joint of the right hand and the second MCP joint of the right hand had no tenderness but was swollen. Nodular thickening of the extensor tendon of the third digit of the right hand was also noted.

**Figure 1:**
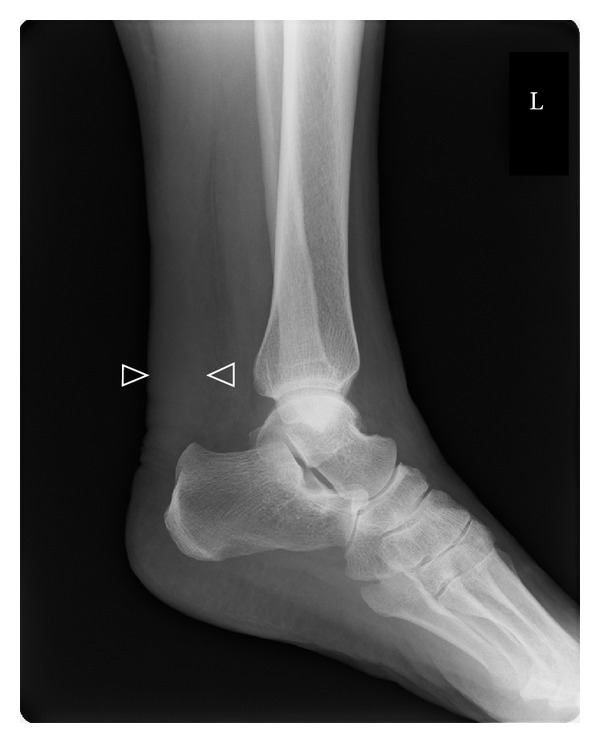
X-ray of the ankle. X-ray of the ankles showed thickening of the bilateral Achilles tendons [left side (arrows heads): 20 mm, right side (not shown): 19 mm].

Laboratory examination revealed an erythrocyte sedimentation rate of 118 mm/h, a C-reactive protein level of 2.06 mg/dL, a rheumatoid factor of 49.3 U/mL (normal <15), an anti-CCP2 antibody level >300 U/mL (normal <4.5), and a matrix metalloproteinase 3 level of 62.3 ng/mL (normal <59.7). X-ray imaging of the hands, feet, ankles, and knees revealed neither bone erosion nor enthesophytes. X-rays of the ankles showed widening of soft tissue shadow suggestive of thickening of the bilateral Achilles tendons (Figure 1).

**Figure 2:**
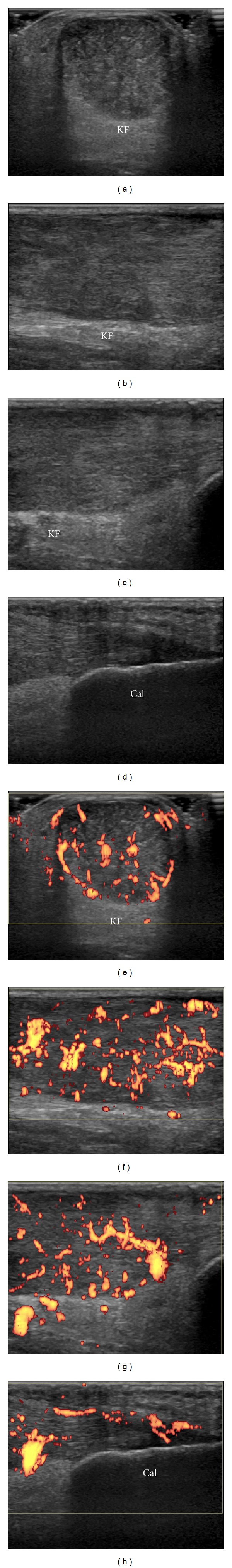
Ultrasound findings of the Achilles tendon. Gray-scale ((a)–(d)) and power Doppler ((e)–(h)) ultrasonograms of the right Achilles tendon (AT). Transverse scans at the midportion ((a), (e)) and longitudinal scans at the midportion ((b), (f)), at the level of retrocalcaneal bursa ((c), (g)), and at the insertion into the calcaneal bone ((d), (h)) of the right AT. Similar findings were also observed in the left AT (not shown). KF Kager's fat pad, Cal calcaneus.

Musculoskeletal ultrasonography (US) disclosed marked multiple tendinitis in the legs and fingers. Gray-scale US (GSUS) images of the Achilles tendon revealed a markedly swollen tendon with a rounded cross section (Figure 2(a)). The tendon appearance was inhomogenous and predominantly hypoechoic with loss of the fibrillar pattern (Figures 2(b)–2(d)). Power Doppler US (PDUS) revealed markedly increased vascularity of the Achilles tendons. Increased blood flow was observed both inside and at the periphery of the tendons (Figures 2(e)-2(h)). The vessels appeared to enter from the ventral aspect of the tendon through the Kager's fat. These abnormal appearances in both GSUS and PDUS were recognized bilaterally and found predominantly along the midportion of the Achilles tendon rather than at the level of the tendon insertion on the calcaneus. No remarkable thickening of the paratenon was observed. Neither bony irregularity of the calcaneus nor retrocalcaneal bursitis was detected. No other ankle lesions, including talocrural joint synovitis, subtalar joint synovitis, tibialis posterior tenosynovitis, or peroneal tenosynovitis, were detected.

**Figure 3:**
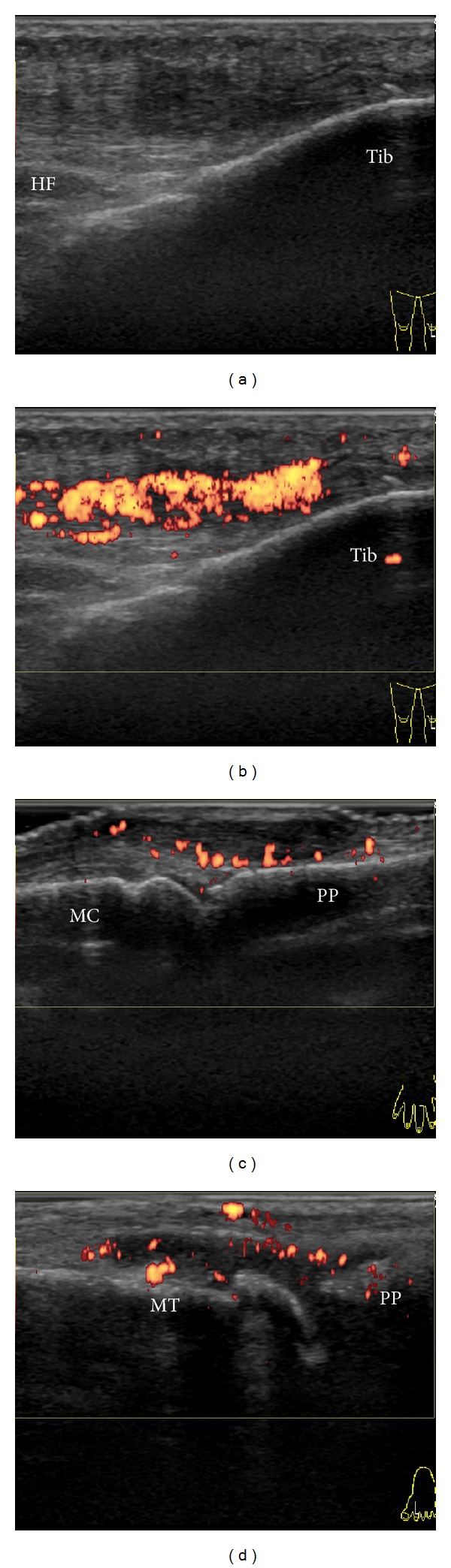
Ultrasound findings of knees, fingers, and toes. (a) Gray-scale and (b) power Doppler ultrasonograms of the longitudinal scan of the distal right patellar tendon (PT). Similar findings were also observed in the left PT (not shown). (c) Power Doppler ultrasonograms of the longitudinal scan of the extensor tendon and metacarpophalangeal joint of the third finger of the left hand. (d) Power Doppler ultrasonograms of the longitudinal scan of the metatarsophalangeal joint of the second toe of the left foot. HF Hoffa's fat pad, Tib Tibia, MC metacarpal bone, PP proximal phalange, MT metatarsal bone.

US findings of the knees were similar to those of the ankles. GSUS revealed focal thickening of the patellar tendon with inhomogenous and hypoechoic appearance and loss of the fibrillar pattern along the distal portion of the tendon (Figure 3(a)). PDUS revealed markedly increased blood flow in the hypoechoic areas inside of the tendon observed by GSUS (Figure 3(b)). These abnormal appearances were recognized bilaterally and found predominantly along the distal portion of the patellar tendon rather than at the level of the tendon insertion on the tibial tuberosity. Neither bony irregularity of the tibia nor infrapatellar bursitis was detected. Except for the patellar tendon lesions, mild synovitis was observed in the bilateral suprapatellar pouch. In the hands, GSUS revealed focal thickening of the finger extensor tendons with hypoechoic appearance and loss of the fibrillar pattern in the dorsal aspect of the right third MCP joint and the left second and fourth MCP joint. PDUS revealed increased blood flow around the hypoechoic areas of the extensor tendons (Figure 3(c)). Except for the extensor lesions, mild synovitis was observed in the right second MCP joint.

The hypoechoic areas detected inside of the Achilles tendons, patellar tendons, and finger extensor tendons presumably correspond to tendon xanthomas with or without tendinosis. The markedly increased blood flow inside and/or around the hypoechoic areas cannot be explained solely by slowly developed neovascularization into the tendon xanthomas. It is evident that there occurred a highly active tendinitis, which is considered a subacute inflammatory process.

In the feet, synovial thickening with hyperemia, which was consistent with RA, was detected in the first, second, third, and fifth MTP joints of the right foot and the first and second MTP joints of the left foot (Figure 3(d)). According to the 2010 RA classification criteria, she was classified as having RA and methotrexate was started [7]. Three months later, swelling and tenderness on palpation of the bilateral Achilles tendons and patellar tendons were resolved, although thickening of the bilateral Achilles tendons persisted.

## 3. Discussion

RA is a chronic inflammatory disease that predominantly affects the synovial membranes lining the joints and tendon sheaths. The affected synovium is proliferative and erosive in character. In addition to synovial tendon sheaths, tendons are also occasionally involved in RA. Our previous study regarding the US findings of Achilles tendon-related involvement in RA found that retrocalcaneal bursitis was the most common pathology and was frequently observed in early RA patients [8]. Achilles paratenonitis was relatively rare and tended to be observed in early RA. As for tendinitis, insertional tendinitis, enthesitis of the Achilles tendon tended to be observed in established RA patients. Noninsertional tendinitis of the Achilles tendon was less common and was almost equally observed between early RA and established RA.

The overall US findings of this case and the degree of each pathology are summarized in Table 1. Although the US examination was performed in the very early phase of RA, the degree of Achilles and patellar tendinosis was high, suggesting that the tendinosis had been present for a long time. The main cause of the tendinosis is thought to be the xanthomas due to genetically determined HeFH rather than the tendinitis due to the newly occurring RA. The patient presented with marked tendinitis in multiple tendons, including the Achilles tendons, patellar tendons, and finger extensor tendons, at the onset of RA. Our examination revealed that tendon lesions were predominantly tendinitis rather than paratenonitis, and that the tendinitis was of the noninsertional variety, rather than the insertional variety. Because this pattern of tendon inflammation is uncommon at the onset of RA, it is speculated that the preexisting tendinosis due to HeFH modified the manifestation of tendon pathology.

In HeFH patients, xanthoma formation inside tendons such as the Achilles tendons and thickening of the tendons are frequently observed. US examination is known to be very useful for evaluating tendon involvement in patients with HeFH, although the descriptions of US findings have varied over the decades. Recently, Tsouli et al. reported the GSUS findings of Achilles tendons among 80 untreated adult patients with HeFH [9]. According to the report, an abnormal echostructure with a diffuse heterogeneous echo pattern was observed in 52.5% of the patients and focal hypoechoic lesions indicating the presence of xanthomas were observed in 10% of the patients.

It has been reported that neovessels inside the tendon xanthomas can be detected by PDUS [10, 11]. As for Achilles tendon xanthomas, PDUS reveals increased vascularity in the “avascular zone” where the blood supply is normally very poor. The flow signals are typically observed along the ventral aspect of the Achilles tendon. It has been confirmed that the PD signals represent neovascularization into the tendon xanthomas by histological observations in the excised specimen of a HeFH patient and in heritable hyperlipidemic rabbit models [11, 12].

Grassi et al. recently proposed that PD signals in patients with early arthritis may be related to increased perfusion of nutritional vessels at the level of either bone-perforating canals and/or the fat pad [13]. Such a phenomenon may imply that preexisting vascularity to joint components functions as a gateway to articular inflammation at the beginning of RA. It would be interesting to speculate that the unusually prominent tendinitis in this patient at the onset of RA was induced by the unusual preexisting hypervascularity in the tendons due to tendon xanthomas.

Is there a causal association between genetically determined HeFH and the development of RA in this patient? An incidental association seems highly plausible because of the absence of literature on their coexistence (there has only been one case report) [6]. It is known, however, that patients with HeFH are prone to rheumatic complaints. Klemp et al. interviewed and examined 48 patients with HeFH and reported that 21% had a history of oligoarthritis and 10% had a history of polyarthritis but none had clinical evidence of RA or other arthropathies [14]. The pretreatment cholesterol levels were significantly higher in HeFH patients with musculoskeletal manifestations than in those without. It was also shown that the musculoskeletal manifestations in HeFH patients improved after receiving lipid-lowering treatment. Artieda et al. have shown that macrophages from HeFH patients exhibit a differential gene expression profile characterized by increased plasma tryptase, TNF-*α*, IL-8, and IL-6 expression [15]. This proinflammatory predisposition of macrophages may contribute to the tendency of HeFH patients to present with musculoskeletal manifestations.

Although we cannot completely deny the possibility, it is unlikely that lipid-lowering agents induced her tendinitis for the following reasons. (1) While it has been reported that most statin-induced tendinopathy occurred within the first year of administration [16], pitavastatin had been maintained on constant dose for two years in the present case. (2) While there is a hypothesis that the rapid lowering of cholesterol can provoke exacerbations of tendinopathy [10], her serum LDL-C had been stable at slightly high levels for six months prior to the onset of tendinitis.

In conclusion, here we report a case of HeFH presenting with marked tendinitis in multiple tendons at the onset of RA. Preexisting tendon xanthomas might contribute to the unusually dominant noninsertional tendinitis of the Achilles tendons, patellar tendons, and finger extensor tendons. Although it was a very rare case, the observation by PDUS throws some light on the role of existing vessels in the initial phase of rheumatoid inflammation.

## Figures and Tables

**Table 1:** The degree of ultrasound pathologies at the onset of rheumatoid arthritis in the present case.

Pathology	Degree
Tendinosis	+++
Noninsertional tendinitis	+++
Paratenonitis	++
Insertional tendinitis (enthesitis)	+
Joint synovitis	+
Tenosynovitis	−
Bursitis	−

## References

[1] Silman AJ, Pearson JE (2002). Epidemiology and genetics of rheumatoid arthritis. *Arthritis Research and Therapy*.

[2] Yamanaka H, Sugiyama N, Inoue E, Taniguchi A, Momohara S (2014). Estimates of the prevalence of and current treatment practices for rheumatoid arthritis in Japan using reimbursement data from health insurance societies and the IORRA cohort (I). *Modern Rheumatology*.

[3] Brown MS, Goldstein JL (1986). A receptor-mediated pathway for cholesterol homeostasis. *Science*.

[4] Goldstein JL, Hobbs HH, Brown MS, Scriven CR, Beaudit AL, Sly WS, Valle D (1995). Familial Hypercholesterolemia. *The Metabolic Basis of Inherited Disease*.

[5] Teramoto T, Sasaki J, Ishibashi S (2014). Familial hypercholesterolemia. *Journal of Atherosclerosis and Thrombosis*.

[6] Josipovic B, Jablanovic D, Josipovic A, Nedeljkovic R, Ilic S (2003). Unrecognized seropositive RA and SS in a patient with associated familial hypercholesterolemia type IIa and osseous xanthoma of the proximal femur. *Clinical and Experimental Rheumatology*.

[7] Aletaha D, Neogi T, Silman AJ (2010). 2010 Rheumatoid arthritis classification criteria: an American college of rheumatology/European league against rheumatism collaborative initiative. *Annals of the Rheumatic Diseases*.

[8] Suzuki T, Okamoto A (2013). Ultrasound examination of symptomatic ankles in shorter-duration rheumatoid arthritis patients often reveals tenosynovitis. *Clinical and Experimental Rheumatology*.

[9] Tsouli SG, Xydis V, Argyropoulou MI, Tselepis AD, Elisaf M, Kiortsis DN (2009). Regression of Achilles tendon thickness after statin treatment in patients with familial hypercholesterolemia: an ultrasonographic study. *Atherosclerosis*.

[10] Abate M, Schiavone C, Salini V, Andia I (2013). Occurrence of tendon pathologies in metabolic disorders. *Rheumatology*.

[11] Ahn JH, Chun TJ, Lee S (2011). Nodular excision for painful localized achilles tendon xanthomas in type II hyperlipoproteinemia: a case report. *Journal of Foot and Ankle Surgery*.

[12] Nakano A, Kinoshita M, Okuda R, Yasuda T, Abe M, Shiomi M (2006). Pathogenesis of tendinous xanthoma: histopathological study of the extremities of Watanabe heritable hyperlipidemic rabbits. *Journal of Orthopaedic Science*.

[13] Grassi W, Filippucci E (2013). Rheumatoid arthritis: diagnosis of RA—we have a dream. *Nature Reviews Rheumatology*.

[14] Klemp P, Halland AM, Majoos FL, Steyn K (1993). Musculoskeletal manifestations in hyperlipidaemia: a controlled study. *Annals of the Rheumatic Diseases*.

[15] Artieda M, Cenarro A, Junquera C (2005). Tendon xanthomas in familial hypercholesterolemia are associated with a differential inflammatory response of macrophages to oxidized LDL. *FEBS Letters*.

[16] Marie I, Delafenêtre H, Massy N, Thuillez C, Noblet C (2008). Tendinous disorders attributed to statins: a study on ninety-six spontaneous reports in the period 1990–2005 and review of the literature. *Arthritis Care and Research*.

